# Amerind Ancestry, Socioeconomic Status and the Genetics of Type 2 Diabetes in a Colombian Population

**DOI:** 10.1371/journal.pone.0033570

**Published:** 2012-04-17

**Authors:** Desmond D. Campbell, Maria V. Parra, Constanza Duque, Natalia Gallego, Liliana Franco, Arti Tandon, Tábita Hünemeier, Cátira Bortolini, Alberto Villegas, Gabriel Bedoya, Mark I. McCarthy, Alkes Price, David Reich, Andrés Ruiz-Linares

**Affiliations:** 1 Department of Genetics, Evolution and Environment, University College London, London, United Kingdom; 2 Laboratorio de Genética Molecular, Universidad de Antioquía, Medellín, Colombia; 3 Department of Genetics, Harvard Medical School, Boston, Massachusetts, United States of America; 4 Departamento de Genética, Universidad Federal do Rio Grande do Sul, Porto Alegre, Brazil; 5 Oxford Centre for Diabetes, Endocrinology and Metabolism, University of Oxford, Churchill Hospital, Oxford, United Kingdom; 6 Wellcome Trust Centre for Human Genetics, University of Oxford, Oxford, United Kingdom; 7 Oxford NIHR Biomedical Research Centre, Churchill Hospital, Oxford, United Kingdom; 8 Department of Epidemiology, Harvard School of Public Health, Boston, Massachusetts, United States of America; 9 Broad Institute of Harvard and MIT, Cambridge, Massachusetts, United States of America; University of Utah, United States of America

## Abstract

The “thrifty genotype” hypothesis proposes that the high prevalence of type 2 diabetes (T2D) in Native Americans and admixed Latin Americans has a genetic basis and reflects an evolutionary adaptation to a past low calorie/high exercise lifestyle. However, identification of the gene variants underpinning this hypothesis remains elusive. Here we assessed the role of Native American ancestry, socioeconomic status (SES) and 21 candidate gene loci in susceptibility to T2D in a sample of 876 T2D cases and 399 controls from Antioquia (Colombia). Although mean Native American ancestry is significantly higher in T2D cases than in controls (32% v 29%), this difference is confounded by the correlation of ancestry with SES, which is a stronger predictor of disease status. Nominally significant association (P<0.05) was observed for markers in: *TCF7L2, RBMS1, CDKAL1, ZNF239, KCNQ1* and *TCF1* and a significant bias (P<0.05) towards OR>1 was observed for markers selected from previous T2D genome-wide association studies, consistent with a role for Old World variants in susceptibility to T2D in Latin Americans. No association was found to the only known Native American-specific gene variant previously associated with T2D in a Mexican sample (rs9282541 in *ABCA1*). An admixture mapping scan with 1,536 ancestry informative markers (AIMs) did not identify genome regions with significant deviation of ancestry in Antioquia. Exclusion analysis indicates that this scan rules out ∼95% of the genome as harboring loci with ancestry risk ratios >1.22 (at P < 0.05).

## Introduction

The public health burden of T2D in Latin America is very high and increasing. About 6% of the adult population in the region (over 16 million people) suffers from the disease and this figure is expected to rise to about 33 million by 2025 [1]–[3]. A number of studies indicate that there is a higher prevalence of T2D in US Native Americans and “Latinos” (or “Mestizos”, i.e. populations mostly of mixed Native American and European ancestry) than in European-Americans, and that disease risk increases with a higher proportion of Native ancestry [4]–[8]. These observations led to the proposal of the ‘thrifty genotype’ hypothesis which posits that metabolic adaptation to a low calorie intake in Native American s has made people with higher Native American ancestry especially prone to a range of metabolic disorders when exposed to a lifestyle including a high calorie diet and little exercise [9–]. Despite the intense interest that this hypothesis has generated its proposed genetic basis remains undefined.

We recently evaluated the genetic ancestry of a sample of T2D cases and controls from a South American mestizo population (Antioquia, Colombia) [12]. Consistent with the existence of Native American-specific genetic susceptibility factors, Antioquian T2D cases show increased Native American ancestry relative to controls from the same population. However, accounting for socioeconomic status rendered this difference in ancestry non-significant, suggesting that environmental factors could at least partially explain the association of Native ancestry and T2D disease risk [12].

To further investigate the basis of T2D susceptibility in Antioquia we carried out a refined assessment of Native American ancestry and SES in an enlarged case/control sample from this population, examined association in this sample to a selection of candidate region SNPs (chosen mostly from recent GWAS) and performed a genome-wide admixture mapping scan aimed at detecting loci carrying Native American T2D susceptibility alleles (as proposed by the thrifty genotype hypothesis). Our results: i) underline the high impact of SES on T2D susceptibility, ii) are consistent with an important role for Old World T2D genetic susceptibility variants in Latino/mestizo populations and iii) fail to provide support for the thrifty genotype hypothesis.

## Results

### Study Sample and Disease Covariates

**Figure 1:**
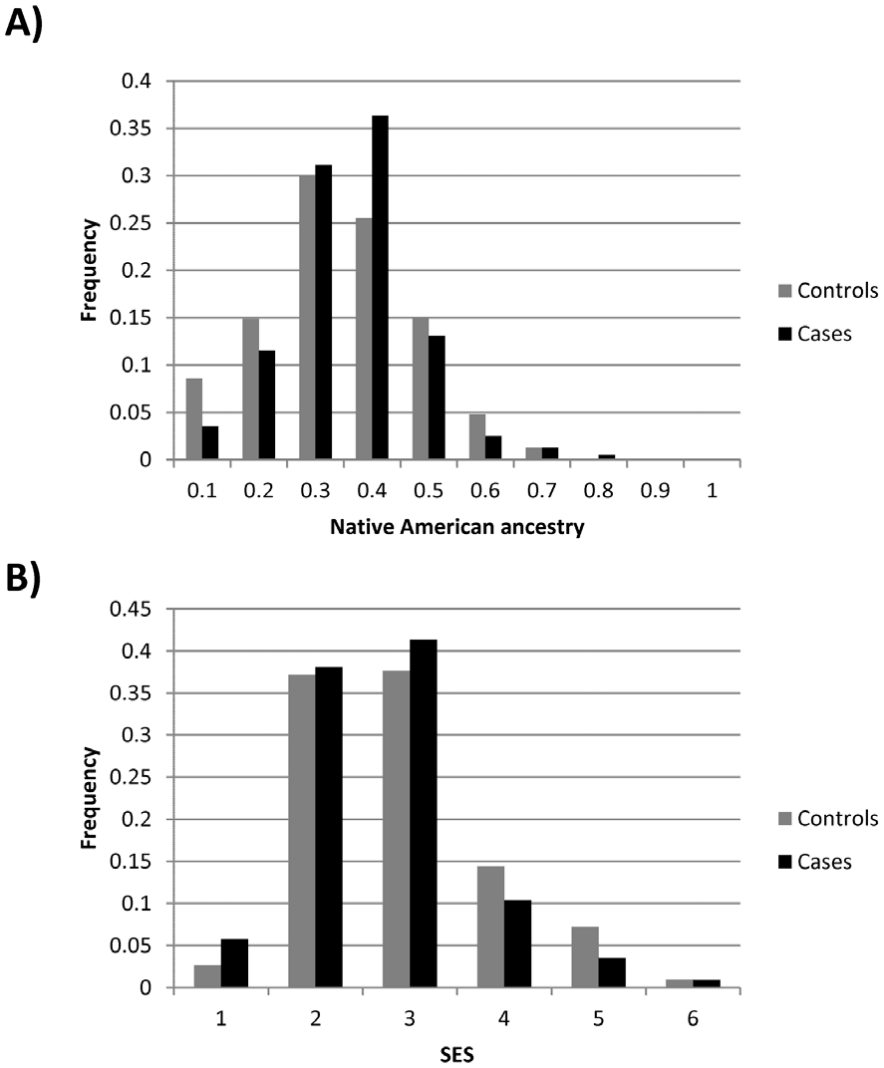
Distribution of (A) Native American ancestry and (B) socioeconomic status (bands 1 to 6) in Antioquian T2D cases and controls.

**Figure 2:**
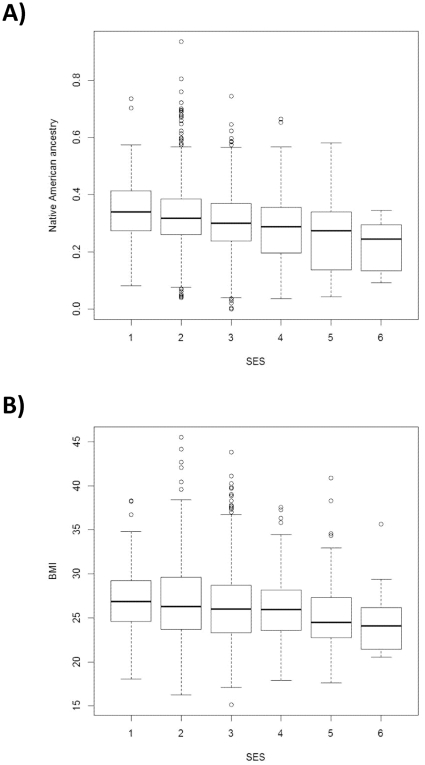
Box plots of (A) Native American ancestry and (B) BMI for socioeconomic status bands 1 to 6 in the Antioquian study sample.

The mean Native American ancestry of Antioquian cases/controls was estimated as 32%/29% resulting in a significant association of ancestry with affection status (*c_LVS1_  = * −0.39 P  =  1.66 × 10^−7^). SES is also strongly predictive of affection status (*c_SES_  = * −0.2 P  =  8.4 × 10^−4^), and shows a significant correlation with Native ancestry: (R  =  −0.19 P  =  6.63 × 10^−11^, Figure 1). When both SES and ancestry are considered together the association of ancestry with disease is no longer significant (*c_LVS1_*  =  0.13 P  =  0.58), while the significance of the disease association with SES increases (*c_SES_*  =  −0.46 P  =  6.75 × 10^−9^). BMI is also strongly correlated with SES (Figure 2) but the association of BMI with disease remains highly significant even after accounting for SES and ancestry (*c_BMI_*  =  0.09241, P  =  1.34 × 10^−6^). One of the markers examined here (rs9939609) is located in the *FTO* gene region and has been robustly associated with BMI in previous studies[7]. The Antioquian sample showed a trend towards higher BMI with increasing number of copies of the previously reported associated allele, although not reaching statistical significance (TT: 26.35, AT:26.74, AA: 26.82; P =  0.14).

### Candidate Marker Association

**Figure 3:**
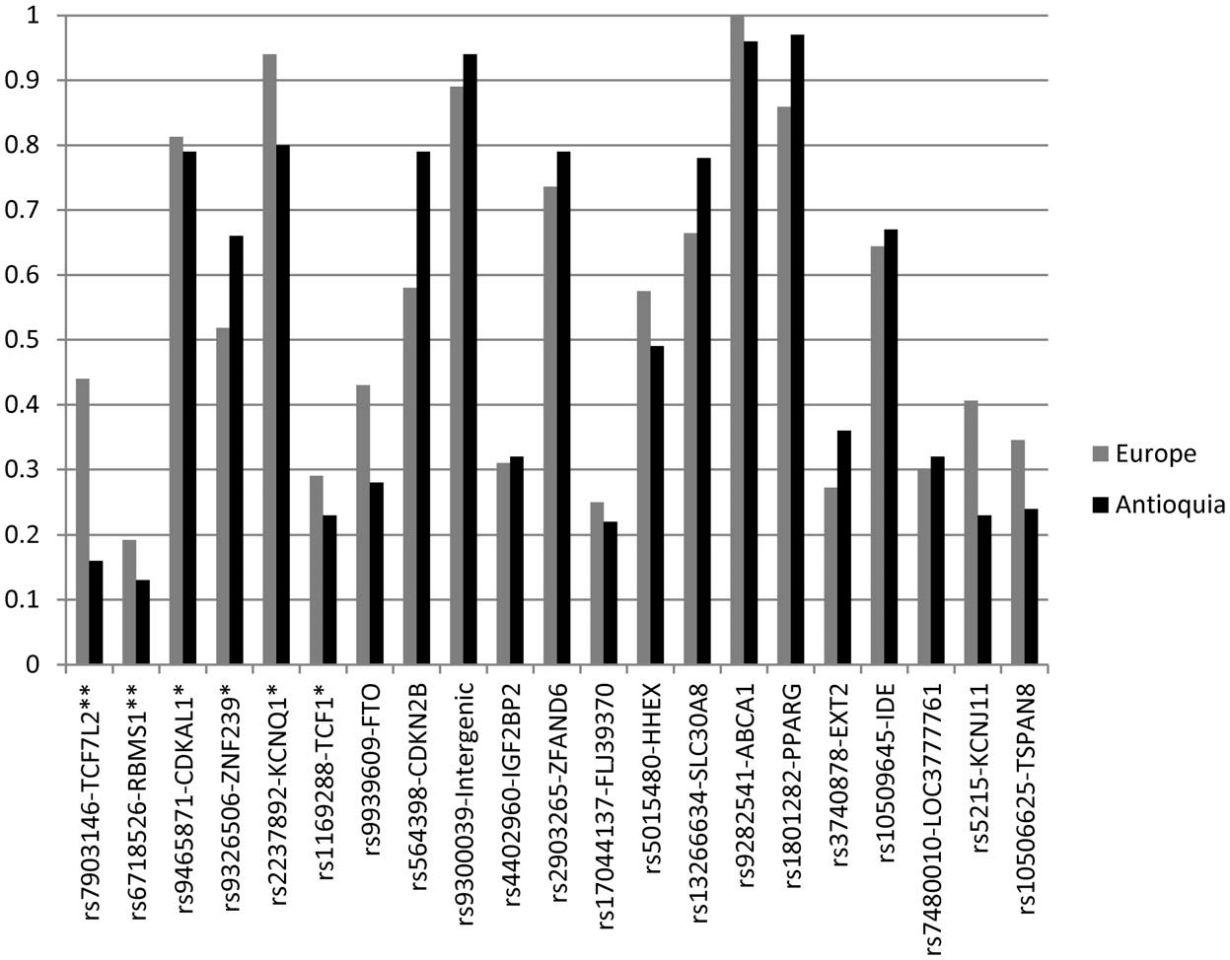
Frequency of the risk allele in Antioquian and in European controls for the marker (-gene region) typed. Markers have been ordered left to right based on the P-value obtained when testing for T2D association in the Antioquian case/control sample (Table 1). Asterisks indicate significance (**<0.01; *<0.05).

None of the markers examined was found to be monomorphic in Antioquia, but three have a reduced polymorphism with minor allele frequencies <5% (rs1801282 in *PPARG*, rs9282541 in *ABCA1* and the intergenic marker rs9300039; Figure 3). Based on the frequencies observed in Antioquian controls (Figure 3) and considering the risk alleles and ORs reported in the literature [13]–[18], the sample examined here has an average power of 41% (range 9–97%) for detecting association at a 5% significance level. Logistic regression incorporating SES, BMI and ancestry as covariates finds nominally significant association (P <0.05) for six of the 21 markers typed (Table 1, Figure 3): rs7903146 (*TCF7L2*), rs6718526 (*RBMS1*), rs9465871 (*CDKAL1*), rs9326506 (*ZNF239*), rs2237892 (*KCNQ1*), and rs1169288 (*TCF1*). All these gene regions, except *ZNF239*, are now considered validated T2D disease loci[19]. P-values at two of these markers exceed thresholds for significance assuming a conservative Bonferroni correction for multiple testing (Table1): rs7903146 (*TCF7L2*) and rs6718526 (*RBMS1*). When considering the 20 markers selected from GWAS hits, 14 show ORs higher than 1 for the previously reported risk allele (Binomial P<0.05). The Native American-specific marker rs9282541 (*ABCA1*) had an OR below 1 for the allele associated with T2D in Mexicans and showed no evidence of significant association in Antioquia (P  =  0.45), despite our sample having 97% power to detect the effect reported in Mexicans[20].

**Table 1:** Association test results for 21 candidate markers (accounting for SES, ancestry and BMI) in Antioquian T2D cases and controls.

Marker	Chromosome	Gene	Risk/Non-Risk Allele^a^	P-value	OR^b^ (95% c.i.)
**rs7903146**	**10**	**TCF7L2**	**T/C**	**0.00012**	**2.28 (1.50, 3.47)**
**rs6718526**	**2**	**RBMS1**	**C/T**	**0.0019**	**1.76 (1.23, 2.51)**
**rs9465871**	**6**	**CDKAL1**	**C/T**	**0.016**	**1.42 (1.07, 1.90)**
rs9326506	10	ZNF239	A/C	0.021	1.32 (1.04, 1.67)
**rs2237892**	**11**	**KCNQ1**	**C/T**	**0.022**	**1.42 (1.05, 1.91)**
**rs1169288**	**12**	**TCF1**	**G/T**	**0.042**	**1.34 (1.01, 1.79)**
**rs9939609**	**16**	**FTO**	**A/T**	**0.062**	**1.30 (0.99, 1.71)**
**rs564398**	**9**	**CDKN2B**	**A/G**	**0.085**	**1.32 (0.96, 1.81)**
**rs9300039**	**11**	**Intergenic**	**C/A**	**0.15**	**1.60 (0.84, 3.03)**
**rs4402960**	**3**	**IGF2BP2**	**T/G**	**0.16**	**1.18 (0.94, 1.50)**
rs2903265	15	ZFAND6	G/A	0.22	0.83 (0.62, 1.12)
rs17044137	4	FLJ39370	A/T	0.23	1.21 (0.89, 1.64)
rs5015480	10	HHEX	C/T	0.23	1.17 (0.90, 1.53)
**rs13266634**	**8**	**SLC30A8**	**C/T**	**0.43**	**0.89 (0.66, 1.19)**
rs9282541	9	ABCA1	A/G	0.45	0.80 (0.45, 1.42)
**rs1801282**	**3**	**PPARG**	**G/C**	**0.48**	**0.76 (0.36, 1.62)**
rs3740878	11	EXT2	A/G	0.51	1.09 (0.85, 1.39)
**rs10509645**	**10**	**IDE**	**C/A**	**0.86**	**0.98 (0.75, 1.27)**
rs7480010	11	LOC3777761	G/A	0.94	0.99 (0.77, 1.28)
**rs5215**	**11**	**KCNJ11**	**C/T**	**0.96**	**0.99 (0.76, 1.31)**
**rs10506625**	**12**	**TSPAN8**	**C/G**	**1**	**1.01 (0.71, 1.41)**

Notes: The gene regions considered established T2D susceptibility loci in Old World populations [19] are shown in bold. (a) Risk allele identified in previous reports.

(b) OR for the previously reported risk allele.

### Admixture Mapping Scan

**Figure 4:**
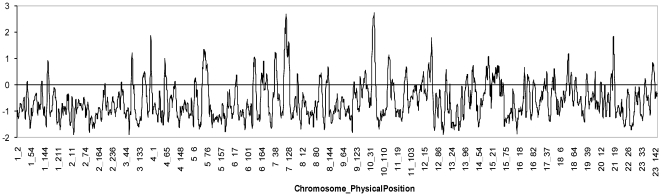
Distribution of LOD-scores for disease association along the genome in the Antioquian T2D admixture mapping scan.

Figure 4 shows the distribution of LOD scores for admixture association in the genome scan of the Antioquian sample. A maximum local LOD-score of ∼2.93 was obtained at positions 109 Mb on chromosome 7 and 58 Mb on chromosome 10. These two peaks do not reach the recommended thresholds for of suggestiveness (LOD-score  =  4) or significance (LOD-score  =  5) of association [21] and are not located at any of the established TD2 susceptibility loci. A genome-wide LOD-score (obtained by averaging the evidence of association at equally spaced points across the genome) of 0.49 was obtained for the full admixture scan, again falling below the recommended cut-off values for suggestive (LOD-score  =  1) or significant (LOD-score  =  2) association. Exclusion analysis indicates that the admixture mapping scan of the Antioquian sample examined here rules out ∼95% of the genome as harbouring loci with ancestry risk ratios >1.22 (at P < 0.05).

## Discussion

This study illustrates some of the complexities of analysing the genetic basis of T2D in Latin American populations, characterized by high genetic and socioeconomic heterogeneity. For historical reasons Latin American individuals and populations show great variability in admixture ratios[22]. Latin America is also a region with high socioeconomic disparities, with Native and highly admixed populations having markedly higher poverty levels than populations with greater European ancestry[23]. The Antioquian population examined here has a history of geographic isolation[24]–[26] and is genetically more homogeneous than many other urban Latin American populations [12], [22]. However, we still observe in Antioquia a highly significant correlation between Native ancestry and socioeconomic status, with higher SES being characterized by lower levels of Native American ancestry. Thus, although we find a significant correlation between Native ancestry and T2D in Antioquia (consistent with other studies [4]–[8], [12], [27], [28] and the thrifty genotype hypothesis) this correlation is strongly confounded by SES. In fact SES is a strong predictor of disease, with lower SES being associated with significantly higher disease risk [12]. There are a number of mechanisms that could explain the impact of SES on T2D risk, such as a less healthy diet and/or lower exercise levels, as suggested by the strong correlation between SES and BMI observed in Antioquia.

Overall, the data for the candidate loci examined are consistent with an important role for T2D variants identified in Old World populations in diseases susceptibility in Antioquia. Nominally significant association was found for nearly half the validated T2D risk loci tested and there is a significant skew towards values >1 of the OR distribution for the markers selected from published T2D GWAS. Most likely the excess frequency of the risk allele at many of the markers tested did not reach statistical significance due to insufficient power to detect the small effects associated with certain variants. Broadly, these results agree with: (i) a role for genetic variants identified in Old World populations in susceptibility to T2D in Latin American populations, (ii) that these variants arose prior to the initial settlement of the Americas and (iii) that they were contributed to admixed Latin Americans both by their Native American and their Old World ancestors. Interestingly, the frequency of the reported risk allele is higher in Europe than in Antioquia at five of the six markers showing nominal significant association (Figure 1), the largest difference (∼20%) being observed for rs7903146 in *TCF7L2*, which is the most robustly replicated T2D susceptibility locus and the one associated with the highest OR (∼1.3) [19]. Typing of control Native American samples confirmed that the difference in allele frequency between Antioquia and Europeans at these loci relates to the admixed ancestry of Antioquia as Native Americans show more extreme allele frequencies at these loci (results not shown). Thus, the difference in risk allele frequency between populations suggests that these susceptibility variants are unlikely to explain the higher disease prevalence in Native Americans and Latinos relative to Europeans. Our observations are consistent with a recent analysis indicating that differences in prevalence between various ethnic groups cannot be accounted for by population differences in risk allele frequencies at established T2D susceptibility loci[29]. Although a substantial fraction of the variation in prevalence across populations is likely to result from environmental (including socioeconomic) differences between them it is possible that unknown population-specific genetic variants also contribute to the increased diabetes prevalence observed in Native Americans and their descendants.

However, we were unable to find evidence of such variants in the Antioquian sample examined here. The admixture scan did not identify regions with significant variation in ancestry along the genome (Figure 1) and rules out ∼95% of the genome as harboring Native American variants of relatively high effect (ancestry risk ratios >1.22). Consistent with the results of this admixture scan, we could not replicate association to T2D for the only known variant private to Native Americans which has been implicated in disease in a Mexican sample (rs9282541 in *ABCA1*) [20]. It is conceivable that improvements in the admixture mapping methodology, the analysis of larger study samples and of populations with greater Native American ancestry than Antioquia might allow the identification of novel, Native American-specific T2D susceptibility loci. However, based on our results, if such loci are identified, they are likely to be responsible for a relatively small increase in disease risk. Our results thus cast some doubt on the thrifty genotype hypothesis as an explanation for increased T2D risk. Recent cross-cultural ethnographic studies have also questioned the validity of the assumptions underlying the thrifty genotype model [30].

In conclusion, our findings underline the importance of socioeconomic status as a confounder in the association of genetic ancestry and T2D risk in Latin American populations. We provide evidence for the involvement of genetic variants identified in the Old World in susceptibility to T2D in Latin America, but find no evidence in support of the thrifty genotype hypothesis.

## Materials and Methods

### Ethics Statement

This research was approved by the BioEthics Committee of Universidad de Antioquia (Colombia), the National Health Service National Research Ethics Service, Central London Committee REC 4 (UK) and the Harvard Medical School Institutional Review Board (USA). Written informed consent was obtained from all participants following the principles of the Declaration of Helsinki. All DNA samples have been anonymized.

### Study Sample

The sample examined here is an expansion of the one analysed in Florez et al. (2009)[12], which consisted of 499 T2D cases and 197 controls typed for 66 AIMs. Here we increased the sample size to 876 T2D cases and 399 controls. Other than the previously typed 66 AIMs, most of the samples were genotyped here for an additional 1,536 AIMs, in order to obtain refined estimates of Native American ancestry and enable an admixture mapping scan. Cases (61% women) were collected from diabetes monitoring clinics in the city of Medellín. Diagnostic criteria included fasting plasma glucose >110 mg/dl or 2-hour glucose >200 mg/dl after a 75-gram oral glucose tolerance test. The mean age of the cases was 63.0 years (SD  =  10.5) and the mean BMI 27.1 (SD  =  4.6). Exclusion criteria included secondary causes of diabetes, genetic syndromes associated with diabetes, and insulin therapy during the first two years after diagnosis. Individuals with no clinical diagnosis of T2D living in the same area as the cases were recruited as controls, restricting selection to individuals over 40 years of age (mean age 60.7, SD  =  10.2) and with no family history of T2D. These strict selection criteria reduced the pool of controls mainly because it was difficult to identify individuals with no family history of diabetes. To ensure local ancestry, we confirmed that at least 6/8 great-grandparents for both cases and controls were born in Antioquia. The main indicator of socioeconomic status used was the banding of the place of residence of individuals assigned by the local government for the purpose of setting the cost of public services (1 being the lowest and 6 the highest).

### T2D Candidate Locus SNP Genotyping

We typed 20 SNPs selected from amongst the most significant association findings of published T2D GWAS [13]–[18]. When several associated SNPs in a gene region with high LD were reported the marker with the smallest P-value was selected for genotyping. Of the 20 gene regions examined 14 are now considered definite T2D susceptibility loci based on recent large-scale meta-analyses [19]. We also typed marker rs9282541 in the *ABCA1* gene. This is a Native American-specific amino-acid changing variant which has been associated with T2D in Mexicans[20]. Markers were genotyped using SNPlex or a competitive allele-specific PCR assay (performed by KBiosciences).

### Ancestry Estimation

A panel of 66 AIMs was genotyped via Sequenom and a panel of 1,536 AIMs was genotyped using the Illumina Golden Gate assay. Details of the markers included in these panels and the genotyping approach have been provided elsewhere [12], [31].The 1,536 AIMs panel is informative mainly for Native American v. Old World ancestry and was specifically designed for admixture mapping in Antioquia [31]. The program EIGENSTRAT [32] was used to identify the main axis of genetic variation. Individuals that were genotyped for the two AIMs panels were used to generate a latent variable (LVS1) as indicator of Native American versus Old World ancestry, which is comparable across the full dataset. This latent variable was derived using structural equation modelling as implemented in the program LAVASE [33].

### Association Testing

Disease association to the candidate SNPs typed was tested via logistic regression, using the R computer package [34], incorporating selected covariates. We denote *c_cov_* to represent the estimate of the regression coefficient for covariate *cov*. Admixture mapping was performed using ANCESTRYMAP [21]. This program calculates a statistic for association at every position in the genome, assuming two parental populations, corresponding to the likelihood of the data at the locus under an average of disease models versus the likelihood of the data if the locus is not associated with the disease. A LOD-score is calculated by taking the log-base-10 of the likelihood ratio. An exclusion map was obtained by establishing the 95% confidence interval for the ancestry risk ratio (*R*) at each location in the genome (defined as increased risk for T2D per copy of Native American ancestry). To obtain the confidence interval we ran ANCESTRYMAP 141 times, in each case testing a single risk model of *R* (from *R*  =  0.2 to R = 3.00 at 0.02 intervals). At each locus, ANCESTRYMAP produced a LOD-score for the tested risk model versus the null model of R  =  1. For each evenly spaced marker in the genome, we identified the maximum likelihood risk model (the one with the highest LOD-score). We then defined the 95% confidence interval for *R* as all other risk models that had a LOD-score within 0.834 of this maximum. This number is justified by the fact that a log-likelihood ratio test states that two times the natural logarithm of the likelihood ratio for the best fitting model to a tested model is expected to have a chi-square distribution with one degree of freedom and thus a value of 3.84 corresponds to a P-value of 0.05 (that is, a log-base-10 of the likelihood ratio equal to 0.834).
